# Cardiotoxicity of Chimeric Antigen Receptor T-Cell (CAR-T) Therapy: Pathophysiology, Clinical Implications, and Echocardiographic Assessment

**DOI:** 10.3390/ijms23158242

**Published:** 2022-07-26

**Authors:** Antonio Nenna, Myriam Carpenito, Camilla Chello, Pierluigi Nappi, Ombretta Annibali, Bruno Vincenzi, Francesco Grigioni, Massimo Chello, Francesco Nappi

**Affiliations:** 1 Cardiac Surgery, Università Campus Bio-Medico di Roma, 00128 Rome, Italy; a.nenna@unicampus.it (A.N.); m.chello@unicampus.it (M.C.); 2 PhD Course, Università Campus Bio-Medico di Roma, 00128 Rome, Italy; c.chello@unicampus.it; 3 Cardiology, Università Campus Bio-Medico di Roma, 00128 Rome, Italy; m.carpenito@policlinicocampus.it (M.C.); f.grigioni@unicampus.it (F.G.); 4 Cardiology, Università degli Studi di Messina, 98122 Messina, Italy; pierluigi.nappi@unime.it; 5 Hematology, Università Campus Bio-Medico di Roma, 00128 Rome, Italy; o.annibali@unicampus.it; 6 Oncology, Università Campus Bio-Medico di Roma, 00128 Rome, Italy; b.vincenzi@unicampus.it; 7 Cardiac Surgery, Centre Cardiologique du Nord de Saint Denis (CCN), 36 Rue des Moulins Gémeaux, Saint-Denis, 93200 Paris, France

**Keywords:** cytokine release syndrome, chimeric antigen receptor T-cell, CAR-T, cardiotoxicity, cardiac, immunotherapy

## Abstract

Contemporary anticancer immunotherapy with chimeric antigen receptor T-cell (CAR-T) therapy has dramatically changed the treatment of many hematologic malignancies previously associated with poor prognosis. The clinical improvement and the survival benefit unveiled the risk of cardiotoxicity, ranging from minimal effects to severe cardiac adverse events, including death. Immunotherapy should also be proposed even in patients with pre-existing cardiovascular risk factors, thereby increasing the potential harm of cardiotoxicity. CAR-T therapy frequently results in cytokine release syndrome (CRS), and inflammatory activation is sustained by circulating cytokines that foster a positive feedback mechanism. Prompt diagnosis and treatment of CAR-T cardiotoxicity might significantly improve outcomes and reduce the burden associated with cardiovascular complications. Clinical and echocardiographic examinations are crucial to perform a tailored evaluation and follow-up during CAR-T treatment. This review aims to summarize the pathophysiology, clinical implications, and echocardiographic assessment of CAR-T-related cardiotoxicity to enlighten new avenues for future research.

## 1. Introduction

Chimeric antigen receptor T-cell (CAR-T) therapy has dramatically improved outcomes of pediatric and adult patients with hematologic disorders [1,2,3]. With the introduction of this treatment, concerns about cardiotoxicity were progressively evaluated. From the mechanistic point of view, the cytokine cascade and signaling mechanisms leading to inflammatory activation are known [4,5,6,7,8], but a better understanding of the macroscopic counterpart is warranted. Cardiac events might result in prolonged hospitalization, life-threatening adverse events, or even death. This review aims to summarize the pathophysiology, clinical implications, and echocardiographic assessment of CAR-T-related cardiotoxicity with particular regards to echocardiography and cardiovascular implications to enlighten new avenues for future research.

## 2. Mechanism of Action

A specific genetic sequence is included in T lymphocytes ex vivo through viral or non-viral vectors and is administered in the systemic circulation of patients who had received lymphodepleting chemotherapy [1,9,10]. Those modified cells preferentially target neoplastic cells with increased expression of specific antigens such as CD19, which is overexpressed in B-cell malignancies (such as acute lymphoblastic leukemia and non-Hodgkin lymphoma). CAR-T formulation requires a patient who has undergone leukapheresis, followed by T-cell selection, activation, and expansion. Therefore, the chimeric antigen receptor is transferred in T cells, refined, and cryopreserved until patient administration. CAR-T therapy has shown dramatic results against hematologic and solid disorders, with a 70–90% response rate in relapsed/refractory conditions [1,7,8,11]. However, early trials suggested that the tremendous cytokine release might have profound clinical implications. As a growing number of patients are treated with CAR-T therapy, a complete understanding of cardiac complications is mandatory as predisposing risk factors (e.g., diabetes) might negatively impact non-oncologic outcomes.

## 3. Pathophysiology of CAR-T-Related Cardiac Damage

After recognizing the tumor antigen, CAR-T cells release proinflammatory cytokines including interleukin (IL)-1, IL-6, the IL-2 receptor, interferon gamma, tumor necrosis factor-alpha, and IL-6 to induce a cytotoxic response [1]. Tissue damage generally results from the activation of cytokine release syndrome (CRS), which is described in most patients and can be directly linked to fatal adverse events. Clinical presentation ranges from mild flu-like symptoms to severe multiorgan failure with capillary leakage with microcirculatory imbalance [1,4,5,6,9,12,13,14,15,16,17,18,19] (Table 1). CRS usually develops after a few days from CAR-T-cell infusion (about two days), with the highest risk of severe reactions during the first two weeks of treatment [4,9,11,12,20]. However, this risk remains theoretically present throughout the treatment, even years after the initial administration [4,12,20]. CRS is reported in about 70–90% of patients, but fortunately, the toxic effects are generally mild [4,9,12]. However, this implies that 10–30% of patients might develop severe/life-threatening complications such as vascular leak syndrome or multiorgan failure [4,9,12,20,21].

In the microscopic scenario, CRS is promoted and sustained by the activation of T cells upon engagement of the CAR by CD19, with the release of proinflammatory mediators that result in macrophage activation and IL-6 production, which sustains the inflammatory process (Table 2). IL-6 also has a direct role in affecting cardiac microvasculature and contraction. The inflammatory milieu promotes capillary leakage, microvascular dysfunction, and increased capillary permeability, exacerbating inflammatory response, which enhances the production of procoagulant factors (such as the von Willebrand factor), causing microvascular obstruction [1,4,5,6,9,12,13,14,15,16,17,18,19].

CRS results in fluid-refractory hypotension and depressed left ventricular function in the macroscopic scenario. Cardiac failure might result in cardiogenic pulmonary edema and might require inotropic support. Cardiomyocyte death is suggested by troponin elevation, ST changes, or stress-induced cardiomyopathy. An inflammatory pericardial reaction might lead to pericarditis of pericardial effusion [1,4,5,6,9,12,13,14,15,16,17,18,19].

## 4. Results of Clinical Investigations

In recent years, many studies have specifically reported the cardiovascular outcomes of CAR-T therapy. However, reporting remains heterogeneous, warranting a standardization of methods and endpoints to allow a detailed comparison in future studies [20,21,22,23,24,25,26,27,28,29,30,31,32,33,34] (Table 3).

Clinically significant hypotension requiring inotropic support or resulting in clinical presentation with cardiogenic shock is generally reported in all studies, ranging from 5% to 30%, with a similar distribution between adult and pediatric patients. Left ventricular dysfunction is reported in 5–10% of patients. Other less common complications are pulmonary edema (4–5%), heart failure (1–6%), and arrhythmias (4–8%). Notably, there is a significant between-studies variability in the incidence of some cardiovascular-related adverse effects, especially considering hypotension and heart failure. This might be partially related to differences in definitions and measured endpoints, which warrants a universal definition of complications and side effects rather than being defined on a single-study basis.

A plethora of cardiac events have been reported in clinical trials. In contrast, in pioneering evaluations, patients with known cardiovascular events were excluded from enrollment, which might have led to underestimating the adverse effect. However, many patients included in those trials had already received therapies with a known increased risk of cardiac events, such as anthracyclines or allogenic stem-cell transplantation [1].

Studies have been performed in both adult and pediatric cohorts, with a mean sample size of about 100 patients in more recent series [4,12,20,21,26,29,33,34]. The most reported and frequent complication is hypotension requiring inotropic support, affecting about 25–30% of pediatric patients and 10% of adult patients [20,21,22,23,24,25,26,27,28,29,30,31,32,33,34]. Left ventricular systolic dysfunction affects 5–10% of patients, but its proper assessment is still debated. More serious complications have rarely been reported (pulmonary edema 5%, cardiac arrest 1–2%), but their occurrence is not negligible. This warrants a specific cardiac assessment before and during CAR-T therapy, with longitudinal outpatient evaluations. Notably, cardiovascular death is reported (and probably noticed) only in studies with an adult cohort of patients. Especially in the adult population, the co-existence of cardiovascular risk factors might exacerbate pre-existing conditions, such as coronary heart disease, that might precipitate with oncologic treatment. Among the pediatric population, CAR-T-related cardiotoxic complications seem to be self-limited, and patients are likely to return to baseline cardiac function even after cardiac arrest. This recovery generally takes 3–6 months, and none of the cardiac events contributed to mortality [1,27,34].

## 5. Baseline Assessment

Baseline factors that might be associated with a higher risk of CRS have been progressively investigated in retrospective evaluation (Table 4) [1,4,12,20,21,22,27,28,29,30,31,34,35]. Intuitively, a high disease burden or a high-intensity lymphodepleting regimen is associated with more severe CRS. Notably, some known factors for cardiotoxicity, such as previous anthracycline treatment, irradiation, or history of stem-cell transplantation, are still not included in the list. This might be due to the constraints of the retrospective evaluation. Risk factors predisposing patients to cardiac adverse events should be carefully investigated before CAR-T therapy. Patients should perform an ECG and a baseline echocardiographic assessment to rule out arrhythmias, valvular heart disease, or coronary artery disease. Optimization of cardiovascular function is recommended before CAR-T therapy, as factors that could lead to heart failure must be treated to avoid life-threatening complications [4,9,12,20].

## 6. Echocardiographic Assessment

Transthoracic echocardiography is commonly used to assess the systolic function of the ventricles and is, therefore, a cornerstone in the diagnosis of cancer-therapy-related myocardial dysfunction (CTRCD). A detailed echocardiographic assessment of parameters involved in CAR-T therapy related to cardiac dysfunction has been performed in a few recent studies [4,12,20,21,27,30,31,34] (Table 5). In both adult and pediatric cohorts, markers of cardiac dysfunction presented a decrease of ≥10% in the left ventricular ejection fraction (LVEF) compared with baseline, a decrease of ≥5% in the left ventricular shortening fraction (LVSF) compared with baseline, and LVEF < 55% or LVSF < 28% in those with previously normal systolic function. A significant decrease in LVEF was described in patients with early mortality for cardiovascular-related issues [21]. Systolic function reduction might be partially reversible after therapy, suggesting that inflammatory edema and microvascular occlusion might be self-limiting in some patients. However, a false-positive diagnosis of CTRCD may occur in up to 3.6% of cancer patients undergoing four longitudinal echocardiographic examinations [35,36]. As is known, the reproducibility of echocardiographic assessments is limited by interobserver and intraobserver variability and/or physiological factors (a high-stress state, the vasomotor effects of anticancer drugs, or the presence of anemia in loading conditions) [37]. If the transthoracic echocardiography image quality is not optimal, additional information can be obtained using cardiac MR. With this method, it is possible to extract data on tissue characterization and the relative presence of edema or fibrosis, which contribute to the diagnosis of cardiac dysfunction. Nowadays, changes in myocardial deformation as determined by strain and strain rate are recognized markers of subclinical CTRCD. Global longitudinal strain was investigated in the pediatric population and might represent an innovative technique for early diagnosis of heart failure with preserved LVEF, but results should be validated in tailored studies.

## 7. Management of CAR-T-Induced Cardiotoxicity

Patients with a CRS grade ≥ 2 are at higher risk for cardiotoxicity during CAR-T therapy and should be carefully monitored [1]. However, no specific guidelines for CAR-T-induced cardiotoxicity are available. A collaborative work is essential to prevent and treat cardiotoxicity without compromising cancer treatment to maximize overall patient outcomes [38]. The management of cardiovascular events is generally based on supportive care, with hemodynamic and respiratory support according to the patient’s cardiovascular and neurologic status. In patients receiving high-dose combination chemotherapy, persistent elevation in cardiac troponin I from a normal baseline may identify those who develop CTRCD with a poor prognosis and who may benefit from treatment with ACE inhibitors [39]. However, there is limited evidence that troponin monitoring is useful for predicting future LV dysfunction with the use of other targeted or immune cancer therapies [38].

Since CAR-T-induced cardiotoxicity is sustained by cytokine activation (mainly IL-6), advanced and peculiar therapies have been gradually introduced in recent years. IL-6 is the crucial component of CRS pathophysiology, leading to capillary leak, vascular dysfunction, complement activation, and myocardial dysfunction [40,41]. Tocilizumab is a monoclonal antibody that inhibits downstream IL-6 signaling by inhibiting IL-6 and its receptor interaction. Tocilizumab received FDA approval for grade 3 and grade 4 CRS, and its effects have been indirectly evaluated by Alvi et al. [21], as a delay in tocilizumab initiation resulted in a higher risk of cardiovascular events. Tocilizumab is the first-line therapeutic agent for moderate-to-severe CRS, generally used in patients with hemodynamic instability or with evidence of end-organ damage (refractory arrhythmias, myocardial infarction, or severe reduction of ventricular function) [4,5,9,12,16,20,21,42]. Siltuximab directly binds to circulating IL-6 and is under clinical evaluation, but might be a promising alternative in patients unresponsive to tocilizumab [1]. A crucial aspect regards whether tocilizumab also inhibits the beneficial effect of CAR-T in specific settings, but at present no reliable data are available.

Considering cytokine activation, the use of corticosteroids seems reasonable in CRS. Corticosteroids are generally administered with tocilizumab in patients with severe CRS [3,9,41]. High doses are used in patients with refractory hypotension, but scientific evidence does not support its efficacy [34]. In fact, in the setting of sustained immune activation with IL-6 production, the level of circulating endogenous corticosteroids is increased by a feedback mechanism [43]. Additional data about corticosteroid use in CAR-T cardiotoxicity are warranted to investigate its therapeutic usefulness.

Considering the clinical benefits of specific anti-IL-6 treatment, recent studies focused on other relevant cytokines involved in CRS. Anakinra, an IL-1 inhibitor available for rheumatoid arthritis, has been associated with reduced cardiac mortality in animal models of severe CRS [44,45] and used clinically in a patient with very severe CRS [46], resulting in significant symptom improvement.

Similarly, anti-TNF antibodies (infliximab) and soluble TNF receptors (etanercept) have been reported to be effective in CRS [35,47,48]. However, clinical validation of IL-1 inhibitors and TNF blockers is awaited in coming studies.

## 8. Conclusions

**Figure 1:**
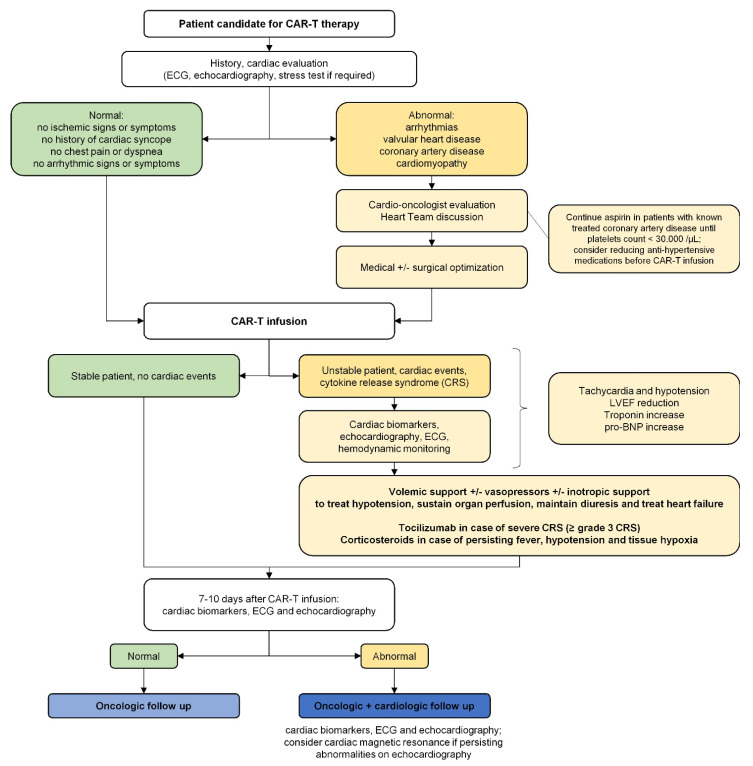
Cardiac evaluation before, during, and after CAR-T therapy. CAR-T: chimeric antigen receptor T-cell; CRS: cytokine release syndrome; LVEF: left ventricular ejection fraction.

Considering the clinical benefit of CAR-T therapy, cardiac disease cannot be considered an absolute contraindication, as oncologic benefits outweigh cardiologic complications and survival significantly improves with this new generation of drugs [1,4,5,6,9,12,13,14,15,16,17,18,19]. However, given the clinical implications and cardiac adverse events associated with CAR-T therapy, a dedicated outpatient evaluation before and during therapy should be recommended, as summarized in Figure 1 based on current recommendations and expert opinions. During CAR-T therapy, the most common complication is hypotension requiring inotropic support, affecting about 25–30% of pediatric patients and 10% of adult patients. Left ventricular systolic dysfunction affects 5–10% of patients, but proper assessment is crucial as cardiac death is a significant concern, especially in the adult population. Therefore, early diagnosis would help to reduce the burden of cardiac-related complications during treatment. In CAR-T-treated patients undergoing outpatient echocardiographic evaluation, current reliable markers of cardiac dysfunction are: a decrease of ≥10% in LVEF compared with baseline, a decrease of ≥5% in LVSF compared with baseline, and LVEF < 55% or LVSF < 28% in those with previously normal systolic function. Changes in left ventricular diameters and volumes are generally included in the evaluation of ejection fraction or shortening fraction and are not used as independent parameters. Global longitudinal strain is a promising marker of early failure but requires tailored validation in dedicated registries. These factors could help detect early cardiac dysfunction during treatment, aiding in the decision of whether to change oncologic treatment or initiate specific cardiac follow-up.

## Figures and Tables

**Table 1:** Cytokine release syndrome (CRS) following CAR-T therapy: grading and clinical presentation.

	Penn Criteria	Lee Criteria	ASTCT Criteria
Grade 1	Mild reaction (supportive care)	Symptoms are not life-threatening and require symptomatic treatment only (fever, nausea, fatigue, headache, myalgias, malaise)	Temperature ≥ 38 °C, no hypotension, no hypoxia
Grade 2	Moderate reaction: signs of organ dysfunction related to CRS and not attributable to any other condition. Hospitalization for management of CRS-related symptoms, including neutropenic fever and need for IV therapies (not including fluid resuscitation for hypotension)	Symptoms require and respond to moderate intervention: oxygen requirement < 40% FiO_2_, hypotension responsive to IV fluids or low dose of one vasopressor	Temperature ≥ 38 °C, with hypotension not requiring vasopressors, and/or hypoxia requiring low-flow nasal cannula
Grade 3	Hospitalization required for management of symptoms related to organ dysfunction. Hypotension treated with multiple fluid boluses or low-dose vasopressors. Coagulopathy requiring fresh frozen plasma, cryoprecipitate, or fibrinogen concentrate. Hypoxia requiring supplemental oxygen (nasal cannula oxygen, high-flow oxygen, non-invasive ventilation)	Symptoms require and respond to aggressive intervention: oxygen requirement ≥ 40% FiO_2_, hypotension requiring high-dose or multiple vasopressors, moderate organ toxicity, or transaminitis	Temperature ≥ 38 °C, with hypotension requiring vasopressors with or without vasopressin, and/or hypoxia requiring high-flow nasal cannula, facemask, nonrebreather mask, or venturi mask
Grade 4	Life-threatening complications such as hypotension requiring high-dose vasopressors. Hypoxia requiring mechanical ventilation	Life-threatening symptoms: requirement for ventilator support, severe organ toxicity	Temperature ≥ 38 °C, with hypotension requiring multiple vasopressors (excluding vasopressin), and/or hypoxia requiring positive pressure (non-invasive ventilation or mechanical ventilation)

**Table 2:** Chimeric antigen receptor T-Cell therapy: from molecular target to clinical manifestation of toxicity [1,4,5,6,9,12,13,14,15,16,17,18,19].

Mechanisms of Action	Toxicity–Molecular Target	Clinical Manifestation of CRS or Cytokines’ Direct Effects **
Autologous T cells are collected via leukapheresis. Lentiviral or retroviral vectors are used to transduce CD-19 specific CARs into T cells. Permanent modification of the genome for long-term gene expression, to recognize and bind specific antigens to the cancer cells. CAR-T cells are grown and multiplied in the bioreactor to create millions of copies. Patient underwent lymphodepletion chemotherapy (*fludarabine 300 mg/mq daily for 3 days and cyclophosphamide 500 mg/mq daily for 3 days*) to suppress the endogenous T-cell compartment. After a quality check, the modified cells are reinfused to the patient where they proliferate, detect, and destroy the tumor cells.	Activation of T cells upon engagement of the CAR by CD19. Release of IL-2, soluble IL-2Ra, IFN-g, and granulocyte–macrophage colony-stimulating factor by the activated T cells and other inflammatory cytokines and chemokines by surrounding immune cells. Increased levels of ang-2, which promotes capillary leakage, along with decreased ang-1, resulting an increased ang-2:ang-1 ratio. IFN-g stimulates macrophages to release IL-6, IL-10, and TNF-a IL-6, and other secreted inflammatory cytokines mediate myocardial dysfunction potentially affecting cardiac integrity. Microvascular dysfunction and increased permeability may further exacerbate cardiac stress and trigger a myocardial inflammatory response, and procoagulant factors, such as vWF, may cause microvascular obstruction. TNF-α has recently been associated with immune-related cardiac dysfunction. ***Cytokine release syndrome (CRS)*** is the most common treatment-related adverse event and is described in 85–93% of patients at any grade. 0–46% experience severe or fatal forms of CRS. Symptoms range from mild flu-like symptoms and fever to life-threatening complications, including capillary leakage, severe hypotension, shock, and multiorgan failure *.	**Cardiotoxicity:** tachycardia; hypotension; fluid refractory hypotension; pulmonary edema; depressed left ventricular function; cardiac failure; cardiac failure requiring inotropic support; elevated troponin; arrhythmia; ST changes; cardiac arrest; stress-induced cardiomyopathy, pericardial disease. **Neurotoxicity:** diminished attention, language disturbance; dysgraphia; confusion; disorientation; agitation; tremors; seizures; motor deficits; increased intracranial pressure; transverse myelitis. **Renal toxicity:** acute kidney injury; electrolyte disturbances. **Hematologic toxicity:** anemia; thrombocytopenia; neutropenia; lymphopenia; DIC; B-cell aplasia; VTE. **Gastrointestinal toxicity:** nausea, vomiting; diarrhea; transaminitis; hyperbilirubinemia.

Ang-1: Angiopoietin 1; 2Ang-2: Angiopoietin 2; CAR: chimeric antigen receptor; DIC: disseminated intravascular coagulation; IL: interleukin; IFN-g: interferon gamma; TNF-α: tumor necrosis factor- alpha; vWF: von Willebrand factor; VTE: venous thromboembolism. * In accordance with a recent consensus approach to grading the severity of cytokine release syndrome, which was released by the American Society for Transplantation and Cellular Therapy (ASTCT) in 2019 [22]. ** Some of the toxicities may in part be attributed to the lymphodepletion regimen used prior to CAR-T-cell infusion and to acute volume changes.

**Table 3:** Summary of reported cardiotoxicity in adult and pediatric populations associated with chimeric antigen receptor T-Cell therapy.

	Maude et al., 2014 [30]	Neelapu et al. (ZUMA-1) [32]	Fitzgerald et al., 2017 [28]	Maude et al. (ELIANA), 2018 [31]	Burstein et al., 2018 [27]	Schuster et al. (JULIET) [33]	Alvi et al., 2019 [21]	Lefebvre et al., 2020 [29]	Shalabi et al., 2020 [34]	Ganatra et al., 2020 [20]	Brammer et al., 2021 [26]
**Patients**	30	101	39	75	98	93	137	145	52	187	102
**Population**	pediatric	adult	pediatric	pediatric	pediatric	adult	adult	adult	pediatric	adult	adult
**Hypotension (Inotropic Support or Shock)**	27%	14%	33%	17%	21%	9%	4%	22.7%	24.3%	2.6%	
**Left Ventricular Systolic Dysfunction**				4%	10%		6%		11.5%	6.4%	
**Pulmonary Edema**				6.7%			4%				
**Cardiac Arrest**		1%		4%					2%		
**Heart Failure**				2.7%			6%	15%		3.2%	1.1%
**Non-fatal Acute Coronary Syndrome**								1.4%			
**Cardiovascular Death**		1%					4%	1.4%		1.6%	
**New or Worsening Arrhythmia**							3.6%	9% (7.6% AFib)		7%	12.2%
**Sinus Tachycardia**		39%		4%		11%	4.4%		69.2%		
**New or Worsening Cardiomyopathy**										10.3%	
**ST-Segment Changes**					6%						
**Biomarker Abnormalities**					NT-proBNP (92%) Lactate (79%) Mixed venous saturation (52%)		Troponin elevation (21%) NT-proBNP (4%)				

**Table 4:** Pre-treatment factors associated with cardiotoxicity during CAR-T therapy.

Hematologic Factors
High disease burden (pre-treatment blasts > 25% on bone marrow biopsy)
High CAR-T dose
Thrombocytopenia
High-intensity lymphodepleting treatment
**Cardiac Factors**
Systolic dysfunction
Diastolic dysfunction
Troponin elevation
Coronary artery disease
Aortic stenosis
**Inflammatory Factors**
Higher C-reactive protein
Hyperlipidemia
**General Factors**
Older age
Higher baseline creatinine

**Table 5:** Echocardiographic parameters involved in chimeric antigen receptor T-Cell therapy-associated cardiac dysfunction.

	Echocardiographic Parameters Linked to Cardiac Dysfunction	Value (Baseline vs. Dysfunction)
**Maude et al. (ELIANA), 2018 (*n* = 75; Pediatric)** [31]	LVSF < 28% by echocardiogram LVEF < 45% by echocardiogram or MUGA	NR
**Burstein et al., 2018 (*n* = 98; Pediatric)** [27]	LVEF decrease of ≥10% or LVSF decrease of ≥5% compared with baseline or LVEF < 55% or LVSF < 28% in those with previously normal systolic function.	NR
**Alvi et al., 2019 (*n* = 137; Adult)** [21]	LVEF decrease > 10% to a value below 50%.	LVEF on the pre-CAR-T echocardiogram was 62 ± 7%, and the LVEDd was 46 ± 6 mm. Twenty nine patients had echocardiographic data pre- and post-CAR-T; of these, eight (28%) had a new reduction in LVEF. A decrease in LVEF from 60% to 19% and from 52% to 32% was described in two patients who died for cardiovascular causes.
**Shalabi et al., 2020 (*n* = 52; Pediatric)** [34]	LVEF > 10% absolute decrease compared to baseline or new-onset left ventricle systolic dysfunction (grade 2, LVEF < 50%). Severe cardiac dysfunction was defined by new-onset LV systolic dysfunction > grade 3 or LVEF < 40%) [23]. GLS was measured retrospectively from previously performed echocardiograms using specific strain software [24].	A total of 6% had an abnormal baseline EF. In contrast, baseline LV GLS was 16.8% (range: 14.1–23.5%, *n* = 37), with 78% (29/37) of patients having a reduced GLS pre-CAR-T-cell infusion (<19%). Six (12%) patients developed cardiac dysfunction (mean range 59% to 30%) including four patients with grade 3–4 CRS. They had concurrent abnormal myocardial strain, with a median LV GLS of 10.1% (range 5.3–14.1%). Four of the six patients had resolution of cardiac dysfunction by day 28 after CAR T-cell infusion. Two patients had persistent cardiac dysfunction with decreased LVEF at day 28 (they received the highest anthracycline exposure before starting CAR-T-cell infusion). One of these patients had the lowest LVEF (10%) during CRS and the other had a slight decrease in LVEF from baseline (50–40%); however, both patients recovered to baseline by the 3-month time point.
**Ganatra et al.,2020 (*n* = 187, Adult)** [20]	LVEF decrease >10% from baseline to <50% during the index hospitalization [25]. Other echo parameters analyzed were: LVEDd (mm); LVESd (mm); LA antero-posterior (mm); and their deviation from baseline.	A total of 12 patients developed new (*n* = 11) or worsening cardiomyopathy (*n* = 1), with a decrease in mean LVEF from 58% to 37% after a mean duration of 12.5 (range, 2–24) days from CAR T-cell infusion. LVEF improved in 9 of 12 patients over a median follow-up of 168.5 days, with normalization to ≥50% in 6 patients and partial recovery in 3 others. All 3 patients in whom LVEF did not recover died: 1 during the index hospitalization from refractory shock and 2 at 189 and 200 days after CAR-T-cell infusion.

CAR: chimeric antigen receptor; GLS: global longitudinal strain; IVS: interventricular septum; LA: left atrium; LVEF: left ventricle ejection fraction; LVEDd: left ventricle end-diastolic diameter; LVESd: left ventricle end-systolic diameter; LVSF: left ventricle shortening fraction; NR: not reported; MUGA: multigated acquisition scan; RVSP: right ventricle systolic pressure.

## Data Availability

Not applicable.
